# Hospital Pharmacists’ Perspectives on Adverse Drug Reaction Reporting in Developed and Developing  Countries: A Comparative Pilot Study

**DOI:** 10.3390/pharmacy13040103

**Published:** 2025-07-29

**Authors:** Javeria Khalid, Tarilate Temedie-Asogwa, Marjan Zakeri, Sujit S. Sansgiry

**Affiliations:** 1 Department of Pharmaceutical Health Outcomes and Policy, College of Pharmacy, University of Houston, Houston, TX 77204, USA; jkhalid4@cougarnet.uh.edu (J.K.);; 2 Boehringer Ingelheim, Ridgefield, CT 06877, USA

**Keywords:** adverse drug reaction, pharmacovigilance, hospital pharmacists, developed and developing country, Pakistan, US

## Abstract

Adverse drug reactions (ADRs) significantly affect patient safety and healthcare spending worldwide. Hospital pharmacists are uniquely positioned to address ADRs due to their crucial role in medication management. However, underreporting remains a global concern, especially in developing countries, where pharmacovigilance systems are inadequately developed. Therefore, this pilot study aimed to evaluate and compare the knowledge, attitudes, perceived barriers, and facilitators regarding ADR reporting by hospital pharmacists in a developed (US) and a developing (Pakistan) country. A cross-sectional survey was conducted, using a pre-validated questionnaire. The pharmacists, possessing a minimum of one year’s hospital experience, were selected via convenience sampling. Out of 151 respondents, included in the final analysis (US: *n* = 51; Pakistan: *n* = 100), the majority were female (62.3%), aged 29–35 years (38%), and possessed a Pharm. D degree (49.7%). The knowledge (US: 6.03 ± 0.27 vs. Pakistan:5.69 ± 0.25, *p*-value = 0.193) and attitude scores (US: 32.02 ± 0.73 vs. Pakistan: 32.63 ± 0.67; *p*-value = 0.379) exhibited no significant differences between the groups. Nonetheless, barriers at both the individual and systemic levels were more pronounced in the developing country. Important facilitators reported were mobile applications for ADR reporting, specialized training, and intuitive reporting tools. In conclusion, we found that pharmacists in both settings exhibit comparable knowledge and positive attitudes towards ADR reporting, though specific contextual barriers are present. Interventions customized to the local hospital infrastructure are crucial for enhancing ADR reporting, particularly in resource-constrained settings.

## 1. Introduction

Adverse drug reactions (ADRs), defined as harmful and unintended responses to a drug at regular doses [1], remain a significant public health issue in modern clinical practice, substantially increasing patient morbidity, mortality, and associated healthcare expenditures [2]. In a recent international analysis of World Health Organization (WHO) mortality data, it was found that global mortality related to adverse drug events (ADEs) has increased more than threefold over the past two decades (2001–2019), reaching 6.86 deaths per 100,000 population, with North America reporting the highest mortality burden [3]. Moreover, recent surveillance statistics of the United States (US) reveal that medication-related injuries constitute around 6.1 emergency department visits per 1000 individuals annually [4]. Approximately 40% of these visits lead to hospitalization, notably with higher rates among older adults [4]. These findings highlight the critical necessity for comprehensive pharmacovigilance (PV) systems capable of efficiently identifying, evaluating, and alleviating drug-related injuries.

Effective ADR monitoring relies heavily on spontaneous reporting systems; yet, worldwide underreporting remains a critical issue, with research indicating that up to 95% of ADRs are unreported [5]. While most high-income countries have made substantial progress in establishing formal PV infrastructures, developing countries remain disadvantaged due to constrained resources, lower awareness, and inadequate reporting systems [6]. Furthermore, a global comprehensive analysis of ADR reporting trends showed that most reports were from developed countries like the US [7]. In contrast, several low- and middle-income countries reported minimal or no ADR submissions to international databases [7]. These observed differences can be attributed to the fact that developed countries already have established systems for ADR reporting [7,8].

Additionally, a recent prospective study also demonstrated that ADRs are of significant concern in hospital settings, with approximately 3.2% of patients experiencing an ADR during their hospital stay and 6.2% being admitted due to an ADR [9]. Furthermore, nearly fifty percent of these ADRs were categorized as serious, with the majority of them were found preventable; this underscores the critical importance of hospital-based ADR monitoring systems [9]. In this context, hospital pharmacists are uniquely positioned among all healthcare professionals to identify and report ADRs due to their direct involvement in drug safety monitoring, antidote administration, and therapeutic management within inpatient settings [10]. Despite this, the ADR reporting rates of hospital pharmacists remain comparatively low [8,11], and data from the FDA Adverse Event Reporting System (FAERS) indicate that pharmacists account for only 6.8% of all ADR reports submitted by healthcare professionals [8,11]. Despite the increasing interest in the responsibilities of pharmacists in pharmacovigilance, current research is mostly limited to single-country contexts and mainly emphasizes community or retail pharmacists [12,13,14,15,16]. Thus, little is known about hospital pharmacists’ ADR reporting behaviors, especially across healthcare systems with varying pharmacovigilance infrastructures and contextual challenges, particularly between developed and developing countries.

Comprehending the contextual barriers and facilitators in each country setting is essential for devising successful interventions that improve PV and patient safety globally. Therefore, this pilot study aimed to evaluate and compare the knowledge, attitudes, and perceived barriers to and facilitators of ADR reporting among hospital pharmacists in a developed country (the United States) and a developing country (Pakistan). For this pilot study, we selected the United States, a high-income country with an established pharmacovigilance infrastructure, and Pakistan, a lower-middle-income country where awareness is growing but reporting rates remain below the WHO recommended threshold of 200 ADR reports per million inhabitants annually [17]. As a result, underreporting of ADR continues to be a significant concern across various regions of Pakistan [18,19].

## 2. Materials and Methods

### 2.1. Study Design, Population, and Study Setting

In this pilot study, a cross-sectional survey, employing questionnaires, was administered between October 2021 and September 2022. This study was designed as an exploratory pilot study to explore the feasibility of using a structured survey instrument to evaluate hospital pharmacists’ knowledge, attitudes, and perceived barriers to ADR reporting across developed and developing countries. The results of this study will serve as a foundation for future large-scale investigations aimed at strengthening pharmacovigilance practices globally.

Enrolled participants were registered hospital pharmacists with ≥1 year of work experience within public or private hospitals within a region of the US or Pakistan. Hospital pharmacists in the US were recruited from the Texas Medical Center (TMC) in Houston, the largest medical complex globally, with over 60 academic and clinical institutions. TMC is globally recognized for its advanced infrastructure, diverse hospital types, and leadership in clinical care and research, offering a comprehensive representation of a high-resource healthcare system. Similarly, participants from Pakistan were recruited from established hospital settings representative of routine clinical practice in a developing healthcare system. Participants were excluded if they were trainees or had less than one year of hospital experience. This study was performed in line with the principles of the Declaration of Helsinki. Ethical approval was obtained from the Institutional Review Board (IRB) before the initiation of the study, and informed consent was acquired from all participants. Additionally, the study was reported in accordance with the STROBE (Strengthening the Reporting of Observational Studies in Epidemiology) checklist to ensure transparency and rigor in reporting.

### 2.2. Questionnaire Instrument

A structured questionnaire was developed for this study by adapting and modifying previously validated instruments from the literature [20,21]. The questionnaire was designed in English and tailored to accommodate the literacy levels and professional contexts of the target healthcare populations in both countries. The design of the questionnaire was informed by the Knowledge, Attitude, and Practice (KAP) model [22]. The final questionnaire consisted of 63 items distributed across five sections.

The first section assessed the knowledge of ADR reporting, using 17 questions on a binary (yes/no) scale. This section was further divided into two parts. The first part consisted of 7 items assessing technical knowledge related to ADR reporting (e.g., definition, regulation, and causality analysis), which required greater conceptual depth and technical accuracy. The second part of the knowledge section included 10 items focused on the participants’ understanding of high-risk populations for experiencing ADRs. The items in the second part were generally more factual, less complex, and more intuitive (Supplementary Table S1). In order to assess respondent attentiveness, reverse scoring questions were incorporated into the knowledge section.

The second section of the questionnaire was comprised of 8 items to assess pharmacists’ attitudes towards ADR reporting by employing a five-point Likert scale (strongly agree, agree, neutral, disagree, and strongly disagree). Similar to the knowledge section, reverse scoring questions in the attitude sections were also considered.

Additionally, the third section was comprised of 18 questions in total, addressing perceived barriers to ADR reporting. This section was further categorized into system-level barriers (8 questions) and individual-level barriers (10 questions). These were also assessed using a five-point Likert scale.

Moreover, the fourth section contained six questions, probing hospital pharmacists’ perspectives on the factors facilitating ADR reporting. For this section also, a five-point Likert scale was used.

The fifth and final section included 14 questions that collected sociodemographic and practice-related information such as age, sex, years of experience, and type of hospital.

### 2.3. Tool Validation

Though the questionnaire used in this study was adapted from previously validated instruments used in PV literature [20,21], however, to ensure content clarity and contextual relevance, the initial draft was validated by 5 researchers and faculty members from the University of Houston, Department of Pharmacy. Similarly, the questionnaire was shared with 3 researchers in Pakistan and adjusted accordingly for readability. Moreover, a preliminary test involving five pharmacists from each country was undertaken to evaluate understanding and suitability. Reliability and internal consistency were assessed using Cronbach’s alpha on the full study sample, resulting in values of 0.70 for the attitude section and 0.89 for the barrier section. This method aligns with conventional methods in survey validation, especially when factor analysis is not performed [23].

### 2.4. Data Collection Process

Data collection employed the Qualtrics Survey software through an anonymous survey system. Participants were recruited using convenience sampling. For the US sample, pharmacists at the TMC were approached via professional networks and institutional email lists. In Pakistan, pharmacists were contacted through academic affiliations, hospital administration lists, and professional associations via email, WhatsApp, and Facebook groups. Participation in the survey was completely voluntary, devoid of any incentives, and respondents had the right to withdraw at any stage of the survey completion process.

### 2.5. Data Analysis

First, the collected data were screened for discrepancies and missing entries, and then, each section was analyzed based on the following scheme. In the knowledge section, to account for differences in cognitive demand, as described in Section 2.2, and to prevent overestimation of knowledge due to the larger number of simpler items in the second part, a differential weighting scheme was applied: each item in Part 1 was scored as 1 point, while each item in Part 2 was weighted as 0.2 points. This approach aligns with the principles from the Classical Test Theory, which recommends adjusting item weights when combining subscales of varying difficulty and relevance to avoid measurement bias [24]. Similar differential weighting strategies have been employed in prior health behavior and KAP studies, especially when combining items of varying complexity or domain specificity [25]. While no universal gold standard exists for weighting in ADR-related KAP surveys, our scoring strategy was guided by content validity considerations, domain experts, and results from the pre-test on 5 hospital pharmacists from each country. Hence, the total attainable knowledge score was 9.

The attitude section involved rating agreements on 8 statements from “strongly disagree (SD)” to “strongly agree (SA)” (1–5 scale). The total score for the attitude section was 40. Moreover, perceived barriers and facilitators were also assessed using a 5-point Likert scale. To identify and rank the most prominent barriers to ADR reporting, the Relative Importance Index (RII) was applied, allowing for the prioritization of both system-level and individual-level barriers within each country.

Data were analyzed descriptively (frequency, percentage, mean, and standard deviation). Bivariate analysis employed chi-square testing for developed and developing country pharmacist comparisons. A multivariable linear regression model was employed to examine the association between knowledge and attitude scores among hospital pharmacists in developed and developing countries, adjusting for sociodemographic and professional practice-related covariates. We used the Mann–Whitney *U* test (Wilcoxon rank-sum test) to examine variations in Likert scale responses regarding perceived barriers and facilitators across groups. A two-tailed *p*-value of less than 0.05 was considered statistically significant.

## 3. Results

### 3.1. Baseline Characteristics

Of the 186 responding hospital pharmacists, 151 were included in the final analysis (US: *n* = 51, Pakistan: *n* = 100). Thirty-five surveys were excluded because five participants did not consent to participate, twenty-nine responses were incomplete, and one survey was improperly filled identified through reverse-coded questions. Moreover, due to the convenience sampling technique and open digital dissemination of the survey, the exact denominator was not available, and therefore, a precise response rate could not be calculated. In our sample, the majority of respondents were female (62.25%) and aged 29–35 years (38%). Respondents with Pharm.D. (Doctor of Pharmacy) degrees were 49.7%, while those with postgraduate degrees (master’s or higher) were 31.1%, with higher proportions in the US (39.22%) compared to Pakistan (27%) (Table 1).

In our selected group, 56.9% of pharmacists were employed in the public/government sector, with a significant majority (83.4%) working full-time. There were a similar number of pharmacists in both countries who mentioned their hospitals had pharmacovigilance/ADR reporting centers (41.2% US vs. 49% Pakistani). Both US (62.75%) and Pakistani (75.0%) pharmacists reported positive support for ADR reporting from managers and colleagues. Training in ADR reporting was lacking for approximately 70.8% of hospital pharmacists, with similar proportions in both developed (64.71%) and developing (74%) countries (*p*-value = 0.23). Nonetheless, a significant majority (89.4%) expressed interest in ADR reporting training, with interest seen in both developed (96%) and developing (86.6%) countries (*p*-value = 0.105) (Table 1). Moreover, a considerable proportion of hospital pharmacist from both countries had not submitted any ADR report in past 6-months (US: *n* = 58.8% and Pakistan: *n* = 49%) (Table 1).

### 3.2. Knowledge of Hospital Pharmacists Regarding ADR Reporting

The overall mean knowledge score among hospital pharmacists was 5.64 ± 1.28 (out of a possible 9). Pharmacists from the US demonstrated a higher mean knowledge score (6.02 ± 1.15) compared to those from the developing country (Pakistan) (5.45 ± 1.31) (Table 1). However, after adjusting for sociodemographic and practice-related variables, multivariable linear regression analysis revealed no statistically significant difference in knowledge scores between the two groups (adjusted means: 6.03 ± 0.27 vs. 5.69 ± 0.25, *p* = 0.1937) (Table 2). Despite the lack of a statistically significant difference in adjusted mean scores, a substantial proportion of pharmacists from Pakistan (48.0%) reported being unaware of international pharmacovigilance organizations (Supplementary Table S1). A significantly greater proportion of pharmacists in the developing country (40.9%) also lacked knowledge regarding the non-requirement of patient consent for ADR reporting, compared to only 5.4% in the US (*p* < 0.001). Interestingly, while only 16.2% of US pharmacists correctly recognized the need for causality assessment prior to ADR reporting, this awareness was notably higher among Pakistani pharmacists (50.4%). In the second section of the knowledge assessment, most respondents from both countries provided correct responses, indicating relatively stronger understanding of the knowledge regarding high-risk populations for experiencing ADRs (Supplementary Table S1).

### 3.3. Attitude of Hospital Pharmacists Regarding ADR Reporting

Hospital pharmacists from both countries demonstrated a generally positive attitude toward adverse drug reaction (ADR) reporting, with mean attitude scores of 32.49 ± 0.37 in the US and 32.51 ± 0.35 in Pakistan, out of a maximum possible score of 40 (Table 1). Furthermore, multivariable linear regression analysis, adjusting for sociodemographic and practice-related covariates, indicated no statistically significant difference in attitude scores between pharmacists from the two countries (adjusted means: 32.02 ± 0.73 for the US vs. 32.63 ± 0.67 for Pakistan; *p*-value = 0.3797) (Table 2).

### 3.4. Perceived Barriers by Hospital Pharmacists Regarding ADR Reporting

Regarding the eight system-based barrier questions, significant disparities in responses emerged for four barriers between the two countries (Table 3). In the developing country, pharmacists expressed concerns about a busy schedule (*p*-value = 0.0166), the unavailability of ADR reporting forms (*p*-value = 0.0002), the absence of an ADR reporting culture (*p*-value = 0.0052), and the lack of a professional body for discussing ADR issues (*p*-value ≤ 0.0001) within their workplaces (Table 4). Analyzing the ten individual-level barriers to ADR reporting, pharmacists from the developing country highlighted primary hindrances, including insufficient knowledge (*p*-value ≤ 0.0001), uncertainty about reporting procedures (*p*-value = 0.0287), and apprehension of retaliation from physicians (*p*-value ≤ 0.0001) (Table 3). The RII underscores the top barriers for US hospital pharmacists, including busy schedules, increased workloads, the complexity of ADR reporting procedures, and concerns about inappropriate forms (Table 4). Conversely, for hospital pharmacists in Pakistan, the highest-ranked barriers encompassed inadequate knowledge for detecting ADRs, lack of a professional body, fear of retaliation from physicians, concerns about additional work, and apprehensions about incorrect form submission (Table 4).

### 3.5. Perceived Facilitators of Reporting ADRs by Hospital Pharmacists

**Figure 1:**
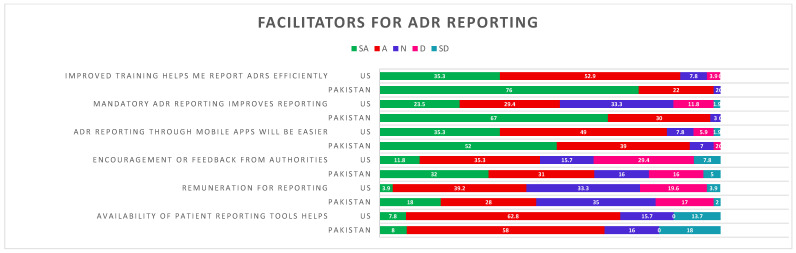
Facilitators for ADR reporting between developed (US) and developing (Pakistan) countries. ADR = adverse drug reaction reporting; SA = strongly agree; A = agree; N = neutral; SD = strongly disagree; D = disagree.

In the developing country, 52% of pharmacists strongly agreed, while in the US, 49% agreed that a mobile app for ADR reporting would enhance the process. A notable majority of pharmacists from both countries (52.9% of US pharmacists and 76% of Pakistani pharmacists) believed that enhanced training and education in ADR reporting could facilitate the process. Additionally, 62.8% of US pharmacists and 58% of Pakistani pharmacists agreed that patient reporting tools could aid ADR reporting. Moreover, a difference in opinion was noted between hospital pharmacists in developed and developing countries regarding the role of higher authorities as facilitators in ADR reporting. Therefore, 67% of Pakistani pharmacists strongly agreed that mandatory reporting would enhance ADR reporting practices, while 33.3% of US pharmacists had a neutral opinion. Similarly, pharmacists from both countries agreed that encouragement or feedback from relevant authorities might enhance ADR reporting, with 32% of Pakistani pharmacists strongly agreeing and 31% agreeing, in contrast to 11.8% and 35.3% among US pharmacists, respectively. However, a larger percentage of pharmacists in the US (29.4%) disagreed with this assertion (Figure 1) (Supplementary Table S2).

## 4. Discussion

In this survey-based study, we examined the knowledge, attitudes, perceived barriers, and facilitators of hospital pharmacists in developed (US) and developing (Pakistan) countries. Notably, no prior comparative study had explored concerns regarding ADR reporting in a developing country compared to a developed country. The interesting findings from this research revealed that there was no significant difference in knowledge scores between the two countries, and both countries’ pharmacists indicated a positive attitude towards ADR reporting. Although the participants exhibited a theoretical understanding of the significance of ADR reporting, their actual implementation was deficient. This indicates a possible disparity between theoretical understanding and the actual competencies necessary for efficient reporting. Moreover, in this study, we analyzed the barriers and facilitators perceived by hospital pharmacists from both developed and developing countries for ADR reporting.

Developed countries have made significant strides in pharmacovigilance, but developing countries still face challenges, with ineffective mechanisms and procedures for reporting ADRs and pursuing drug safety [6]. Although we observed no statistically significant difference in the knowledge score of pharmacists from the two countries, the overall mean knowledge score was modest. Additionally, a pressing need exists for enhanced education ADR reporting process, as a notable majority of pharmacists (52%) from both developed and developing nations concede the non-reporting of ADRs in the preceding six months, presumably due to a deficiency in ADR reporting procedure understanding. This finding aligns with the existing literature, which suggests that insufficient training in ADR reporting is strongly associated with limited knowledge of PV principles and ADR reporting practice [26]. Notably, Pakistani pharmacists showed a noticeable lack of knowledge of international PV organizations and the need for patient consent when reporting ADRs. Studies conducted in Nigeria and India also found that pharmacists there were not well-versed in the worldwide standards for ADR reporting; this further supports the idea that organized PV training is necessary [27,28]. Hospital pharmacists in both countries showed a positive attitude regarding ADR reporting, with no discernible variations in attitude ratings. This finding is in line with research conducted in Malaysia and Saudi Arabia, where pharmacists who expressed a strong willingness to engage in ADR reporting [29,30].

Our investigation has elucidated various factors hindering ADR reporting by hospital pharmacists across both developed and developing countries. Hospital pharmacists from Pakistan were more likely to cite system-related barriers, including a busy schedule, unavailability of ADR reporting forms, a deficit in familiarity with the reporting protocols, the absence of a culture for ADR reporting, and the lack of a professional body for ADR discourse. These issues have been persistently documented in underdeveloped nations, where insufficient institutional support and resource limitations obstruct efficient pharmacovigilance methods [30,31]. Similarly, a study conducted in Pakistan identified pivotal barriers among healthcare professionals, inclusive of an inadequate environment for ADR discourse and insufficient incentives to promote reporting [32]. Prior research has also identified a paucity of workforces, temporal constraints, and insufficient training as a barrier to suboptimal ADR reporting practices among pharmacists [7,33], aligning harmoniously with our study’s findings. Conversely, the US pharmacists predominantly recognized time limitations, substantial workloads, and the intricacies of reporting protocols as significant obstacles, which are also well-documented issues in high-income settings [34]. Furthermore, the Pakistani pharmacists reported uncertainty regarding reporting protocols and fear of physician reprisals as individual-level barriers, which is consistent with earlier reports from other developing countries [12,35]; however, we did not find these to be a concern for hospital pharmacists from the US. The apprehension of professional consequences persists as a considerable obstacle in several healthcare systems and may discourage pharmacists from reporting ADRs, highlighting the necessity for institutional protection to encourage ADR reporting. The relative importance index further highlighted disparities in the rating of perceived barriers for ADR reporting by both countries. Both groups recognized workload and reporting complexity as the most significant barriers. Additionally, Pakistani pharmacists emphasized other high-ranking concerns, including insufficient knowledge, lack of a professional organization, apprehension regarding physician retaliation, and anxiety over erroneous form submissions., These findings indicate the need for both educational and systemic reforms to improve ADR reporting in developing country like Pakistan.

Additionally, the facilitators of ADR reporting varied across the two countries. Pharmacists in both nations robustly endorsed the development of a mobile application for ADR reporting and advocated for more training and educational opportunities. Previous studies corroborate this conclusion, indicating that digital ADR reporting systems can improve reporting efficiency and engagement [36]. The prospective advantages of mobile-based ADR reporting tools have been examined in several contexts, including the WHO’s Vigiflow system, which has enhanced spontaneous ADR reporting rates in numerous countries [37]. Significant disparities were noted with compulsory reporting regulations and institutional support [37]. A considerable percentage of Pakistani pharmacists strongly agree that enforcing ADR reporting would enhance reporting rates, in contrast to the neutral responses from US pharmacists. Comparable patterns have been noted in previous research, when healthcare practitioners in developing nations preferred mandatory reporting systems owing to the absence of a well-established reporting culture [19,38]. Hence, both perceived barriers to and facilitators for ADR reporting indicate that hospital pharmacists in Pakistan perceive system-level deficiencies as significant impediments to ADR reporting. Together with their substantial consensus about the value of regulatory encouragement and mandatory reporting, our findings highlight the need for system-level changes and organized regulatory assistance to improve ADR reporting procedures in developing countries.

A major strength of this pilot study is the comparative analysis of hospital pharmacists perspective from both developed and developing countries, offering useful insights into the contextual disparities affecting ADR reporting. Secondly, though it was a pilot study, the significant findings from this study will serve as a foundation for future large-scale investigations aimed at strengthening PV practices globally. However, our study also has some limitations. First, as a pilot study, the sample size was relatively small, particularly for the US cohort (*n* = 51), which may limit the generalizability of our findings. Moreover, the US participants were all recruited from hospitals affiliated with the TMC in Houston, and while these institutions represent a range of hospital types, including public, private, and nonprofit facilities within a single healthcare complex, this geographic concentration may limit the generalizability of our findings for pharmacists practicing in other US regions. Notably, a substantial proportion of US respondents indicated postgraduate qualifications (e.g., MS/PhD or similar), and while this may appear higher than the national pharmacist averages, it may reflect the evolving professional standards in hospital settings, where residency training (PGY1/PGY2) is increasingly expected and may have been interpreted by respondents as postgraduate credentials [39,40]. This contextual factor should be considered while interpreting the generalizability of our findings to a larger group of hospital pharmacists. Moreover, due to convenience sampling and open digital dissemination of the survey, the exact denominator was not available to estimate a precise response rate. Furthermore, the study depended on self-reported data, which may be influenced by social desirability bias. Future studies should investigate longitudinal interventions to evaluate the effects of tailored training programs and digital reporting tools on ADR reporting behavior of developed and developing countries. Despite these limitations, the researchers are confident in the reliability of the findings, which can assist authorities in enhancing PV activities in the future. This pioneering study compares the knowledge, attitudes, perceived barriers, and facilitators of hospital pharmacists regarding ADR reporting between developed and developing countries, offering valuable baseline data for further research. The results indicate that system-level support is critically important to enhance ADR reporting practices in developing countries. Furthermore, fostering collaboration among regulatory authorities, hospital administration, pharmacists, and other healthcare professionals is essential for raising ADR reporting awareness.

## 5. Conclusions

In this pilot study of hospital pharmacists’ perspectives regarding ADR reporting, we found that, while knowledge and attitudes were comparable between pharmacists in the US and Pakistan, notable differences were identified in the barriers perceived by pharmacists in a developing country compared to their counterparts in the developed country.setting. These findings highlight the need for a system-level approach to enhance ADR reporting practices among hospital pharmacists in developing countries. The insights from this study are intended to inform the development of context-specific guidelines and policies aimed at strengthening pharmacovigilance and drug safety reporting systems, using the US as a reference model for comparison.

## Figures and Tables

**Table 1:** Basic characteristics of hospital pharmacists in developed (US) and developing countries.

	Total (*n* = 151)	US (*n* = 51)	Pakistan (*n* = 100)	*p*-Value
Sex				
Male	57 (37.7)	11 (21.6)	46 (46.0)	0.0034
Female	94 (62.3)	40 (78.4)	54 (54.0)	
Age (Years)				
≤28 Years	55 (36.4)	13 (25.5)	42 (42.0)	<0.0001
29–35 Years	58 (38.4)	11 (21.6)	47 (47.0)	
>35 Years	38 (25.1)	27 (52.9)	11 (11.0)	
Type of hospital				
Government/Public	86 (56.9)	32 (62.8)	54 (54.0)	0.4887
Private	48 (31.7)	13 (25.5)	35 (35.0)	
Other †	17 (11.3)	6 (11.8)	11 (11.0)	
Working hours				
Full-time (more than 20 h)	126 (83.4)	43 (84.3)	83 (83.0)	0.8373
Part-time (less than 20 h)	25 (16.5)	8 (15.7)	17 (17.0)	
Academic degree				
B. Pharm	29 (19.2)	10 (19.6)	19 (19.0)	0.2536
Pharm. D	75 (49.7)	21 (41.2)	54 (54.0)	
Masters, Ph.D., or similar	47 (31.1)	20 (39.2)	27 (27.0)	
No. of years practiced				
1–3 Years	52 (34.4)	21 (41.2)	31 (31.0)	0.2512
4–6 Years	26 (17.2)	11 (21.6)	15 (15.0)	
7–9 Years	23 (15.2)	5 (9.8)	18 (18.0)	
≥10 Years	50 (33.1)	14 (27.5)	36 (36.0)	
Have received ADR training/certificate				
No	107 (70.9)	33 (64.7)	74 (74.0)	0.2346
Yes	44 (29.1)	18 (35.3)	26 (26.0)	
Is there a pharmacovigilance center/ADR reporting center in your hospital?				
No	66 (43.7)	25 (49.0)	41 (41.0)	0.6237
Yes	70 (46.7)	21 (41.2)	49 (49.0)	
Not sure	15 (9.93)	5 (9.8)	10 (10.0)	
Is there a drug safety or medication safety officer in your hospital?				
No	68 (45.0)	26 (50.9)	42 (42.0)	0.3216
Yes	66 (43.7)	18 (35.3)	48 (48.0)	
Not sure	17 (11.3)	7 (13.7)	10 (10.0)	
Are ADRs actively reported by colleagues and other health professionals in your hospital?				
No	77 (50.9)	32 (62.8)	45 (45.0)	0.0391
Yes	74 (49.0)	19 (37.6)	55 (55.0)	
Do hospital managers and your colleagues consider your ADR reporting positively?				
No	43 (28.5)	19 (37.3)	24 (24.0)	0.0878
Yes	108 (71.5)	32 (62.8)	76 (76.0)	
No. of ADRs reported in the last 6 months				
None	79 (52.3)	30 (58.8)	49 (49.0)	0.2530
≥1 ADRs	72 (47.7)	21 (41.2)	51 (51.0)	
Knowledge *	5.64 ± 1.28	6.02 ± 1.15	5.45 ± 1.31	0.0085
Attitude **	32.50 ± 3.30	32.49 ± 0.37	32.51 ± 0.35	0.9697

† Others include public/private partnership hospitals, mixed, trust-based and nonprofit hospitals, and military hospitals. * The maximum possible knowledge score was 9. ** The maximum possible attitude score was 40.

**Table 2:** Results of linear regression analysis of knowledge and attitude scores of ADR reporting by hospital pharmacists in the US and Pakistan.

	US (*n* = 51)		Pakistan (*n* = 100)		Adjusted Difference Between Means *	*p*-Value ^1^
	Mean ± SE	95% CI	Mean ± SE	95% CI	Mean (95% CI)	
Knowledge	6.03 ± 0.27	5.49–6.58	5.69 ± 0.25	5.19–6.19	0.34 (−0.17–0.86)	0.1937
Attitude	32.02 ± 0.73	30.56–33.47	32.63 ± 0.67	31.30–33.97	0.62 (−2.01–0.77)	0.3797

* Adjusted for baseline characteristics defined in Table 1. Sex, age, type of hospital, working hours, academic degree, no. of years practiced, ADR training/certificate, pharmacovigilance center in the hospital, drug safety officer in the hospital, active reporting of ADRs by colleagues, and positive consideration of ADR reporting by managers and colleagues. ^1^ *p*-value ≥0.05 was defined as significant.

**Table 3:** Barriers perceived by hospital pharmacists from the US and Pakistan in reporting ADRs.

	US (*n* = 51)	Pakistan (*n* = 100)	*p*-Value
**System-Based Barriers**	**Median (Q1–Q3)**	**Median (Q1–Q3)**	
Your schedule is too busy to fill out the form.	4 (3–5)	4 (3–4)	**0.0166**
Your practice is too busy to actively look for ADRs.	4 (2–4)	3.5 (2–4)	0.7734
Non-availability of a reporting form at the workplace.	2 (2–4)	4 (2–4)	**0.0002**
Complex procedure of ADR reporting.	4 (2–4)	3 (2.5–4)	0.864
It’s too difficult to identify ADRs in clinical practice.	2 (2–4)	3 (2–4)	0.4552
Fear of legal liability for the reported ADRs.	3 (2–4)	3 (2–4)	**0.0269**
Lack of ADR reporting culture in my hospital.	3 (2–4)	4 (2–4)	**0.0052**
Lack of professional setup/body to discuss ADRs.	3 (2–4)	4 (3–5)	**<0.0001**
**Individual base barriers**			
Apprehension about sending inappropriate forms	3 (2–4)	3 (3–4)	**0.0291**
Concern that extra work is required to fill and send the report	4 (3–4)	4 (3–4)	0.1656
Insufficient knowledge of pharmacotherapy in detecting ADR	2 (2–4)	4 (3–4)	**<0.0001**
I sometimes get confused about what exactly I should report	2 (2–2)	2 (2–3)	**0.0084**
Feeling that reporting of previously known ADR is not required	3 (2–4)	3 (2–4)	0.2634
Not knowing how to report ADRs	2 (2–4)	3 (2–4)	**0.0287**
There is fear of retaliation from physicians when ADR is reported for their prescriptions	2 (2–4)	4 (3–5)	**<0.0001**
I am sometimes not sure if the drug caused the ADR given	2 (2–2)	2 (2–3)	0.0915
Punishment if ADR occurs from your recommendation	3 (2–4)	3 (2–4)	0.7349
Feeling that sending a single report of the patient may not contribute a lot of patient care	2 (2–4)	3 (2–4)	0.074

Note: Bolded *p*-values indicate statistical significance at *p* < 0.05.

**Table 4:** Barriers ranked according to importance for each country.

**US**	RII *	**Ranking**
Your schedule is too busy to fill out the form	0.76	1
Concern that extra work is required to fill and send the report	0.75	2
Complex procedure of ADR reporting	0.67	3
Your practice is too busy to actively look for ADRs	0.65	4
Apprehension about sending inappropriate forms	0.62	5
**Pakistan**	RII *	**Ranking**
Insufficient knowledge of pharmacotherapy in detecting ADR	0.77	1
Lack of professional setup/body to discuss about ADRs	0.72	2
There is fear of retaliation from physicians when ADR is reported for their prescriptions	0.71	3
Concern that extra work is required to fill and send the report	0.70	4
Apprehension about sending inappropriate forms	0.69	5

* RII = Relative Importance Index.

## Data Availability

The data sets generated during and/or analyzed during the current study are available from the corresponding author upon reasonable request.

## Supplementary Materials

The following supporting information can be downloaded at https://www.mdpi.com/article/10.3390/pharmacy13040103/s1: Table S1: Frequency distribution of knowledge questions between hospital pharmacists of developed and developing countries. Table S2: Facilitators for ADR reporting.

## Abbreviations

The following abbreviations are used in this manuscript:

ADR	Adverse drug reactions
US	United States
PV	Pharmacovigilance
FDA	Food and Drug Administration
FAERS	FDA Adverse Event Reporting System
WHO	World Health Organization
STROBE	Strengthening the Reporting of Observational Studies in Epidemiology
KAP	Knowledge, Attitude, and Perception
SA	Strongly agree
SD	Strongly disagree
A	Agree
N	Neutral
D	Disagree
RII	Relative Importance Index
