# Supplementary material for: Hospital Pharmacists’ Perspectives on Adverse Drug Reaction Reporting in Developed and Developing Countries: A Comparative Pilot Study
Source: Pharmacy (Basel). 2025 Jul 29;13(4):103. doi: 10.3390/pharmacy13040103 (PMC12389004; doi:10.3390/pharmacy13040103)
Supplement: Supplementary file 1 [file pharmacy-13-00103-s001.zip › pharmacy-3738288-supplementary.pdf]

Supplementary material

Hospital Pharmacists' Perspectives on Adverse Drug Reaction Reporting in Developed and Developing Countries:

A Comparative Pilot Study

	Supplementary Table S1. Frequency distribution of Knowledge Questions between hospital pharmacists of Developed and Developing country							
		**Total (n = 151)**	**%**	**US (n = 51)**	**%**	**Pakistan (n= 100)**	**%**	**P-value**
	**Part 1: Technical knowledge related to ADR reporting**							
1.	An Adverse Drug Reaction (ADR) is a response to a drug which is noxious, unintended and occurs at normally prescribed FDA approved doses used for the prophylaxis, diagnosis or treatment of disease, or for modification of physiological function.							
	No	8	5.3	1	1.96	7	7	0.2673
	Yes	143	94.7	50	98.04	93	93	
2.	Only the name and dose of a suspected drug needs to be reported on the ADR form, or to the drug safety officer							
	No	50	33.11	11	21.57	39	39	0.0314
	Yes	101	66.89	40	78.43	61	61	
	**Supplementary Table S1. Frequency distribution of Knowledge Questions between hospital pharmacists of Developed and Developing country**							
		**Total (n = 151)**	**%**	**US (n = 51)**	**%**	**Pakistan (n= 100)**	**%**	**P-value**
3.	It is necessary to do causality analysis of ADRs with the offending drug before reporting.							
	No	81	53.64	42	82.35	39	39	<.0001
	Yes	70	46.36	9	17.65	61	61	
4.	It is necessary to obtain consent from the patients before reporting an ADR.							
	No	54	35.76	7	13.73	47	47	<.0001
	Yes	97	64.24	44	86.27	53	53	
5.	The timeframe within which a serious ADR should be reported is 7 days.							
	No	106	70.2	39	76.47	67	67	0.2288
	Yes	45	29.8	12	23.53	33	33	

6.	ADR tracing method through antidotes, antihistamines or steroids (i.e., a trigger tool method) is an ineffective way for hospital ADR reporting							
	No	92	60.93	25	49.02	67	67	0.0322
	Yes	59	39.07	26	50.98	33	33	
7.	FDA is the only body who monitors ADRs internationally							
	No	69	45.7	13	25.49	56	56	0.0004
	Yes	82	54.3	38	74.51	44	44	
	**Part 2: Knowledge regarding high-risk populations for experiencing ADRs**							
8.	Old age greater than or equal to 65 years							
	No	37	24.5	2	3.92	35	35	<.0001
	Yes	114	75.5	49	96.08	65	65	
9.	Children less than 4 Years							
	No	47	31.13	8	15.69	39	39	0.0034
	Yes	104	68.87	43	84.31	61	61	
10.	Gender being male							
	No	40	26.49	20	39.22	20	20	0.0114
	Yes	111	73.51	31	60.78	80	80	
11.	Polypharmacy							
	No	9	5.96	0	0	9	9	0.0288
	Yes	142	94.04	51	100	91	91	
	**Supplementary Table S1. Frequency distribution of Knowledge Questions between hospital pharmacists of Developed and Developing country**							
		**Total (n = 151)**	**%**	**US (n = 51)**	**%**	**Pakistan (n= 100)**	**%**	**P-value**
12.	Pregnancy							
	No	22	14.57	9	17.65	13	13	0.444
	Yes	129	85.43	42	82.35	87	87	
13.	Lactation							
	No	19	12.58	8	15.69	11	11	0.4115
	Yes	132	87.42	43	84.31	89	89	
14.	Drinking Alcohol							
	No	12	7.95	5	9.8	7	7	0.5469
	Yes	139	92.05	46	90.2	93	93	
15.	Smoking							
	No	30	19.87	14	27.45	16	16	0.0953

	Yes	121	80.13	37	72.55	84	84	
16.	Multiple Co-morbidities							
	No	11	7.28	3	5.88	8	8	0.7508
	Yes	140	92.72	48	94.12	92	92	
17	Renal/Hepatic dysfunction							
	No	9	5.96	0	0	9	9	0.0288
	Yes	142	94.04	51	100	91	91	

Supplementary Table S2. Facilitators for ADR reporting

	SA	A	N	D	SD	P-value
**ADR reporting through mobile apps will be easier**						
US	18 (35.29)	25 (49.0)	4 (7.84)	3 (5.9)	1 (1.96)	0.0229
Pakistan	52 (52)	39 (39)	7 (7)	2 (2)	0 (0)	
**Improved training and education might help me to report ADRs efficiently**						
US	18 (35.3)	27 (52.9)	4 (7.84)	2 (3.92)	0 (0)	<.0001
Pakistan	76 (76)	22 (22)	2 (2)	0 (0)	0 (0)	
**The availability of patient reporting tools might help with ADR reporting**						
US	4 (7.84)	32 (62.8)	8 (15.7)	0 (0)	7 (13.7)	0.5264
Pakistan	8 (8)	58 (58)	16 (16)	0 (0)	18 (18)	
**Mandatory ADR reporting will improve the rate of reporting**						
US	12 (23.5)	15 (29.4)	17 (33.3)	6 (11.8)	1 (1.9)	<.0001
Pakistan	67 (67)	30 (30)	3 (3)	0 (0)	0 (0)	
**Remuneration for reporting**						
US	2 (3.9)	20 (39.2)	17 (33.3)	10 (19.6)	2 (3.9)	0.1783
Pakistan	18 (18)	28 (28)	35 (35)	17 (17)	2 (2)	
**Encouragement or feedback from relevant authorities**						
US	6 (11.8)	18 (35.3)	8 (15.7)	15 (29.4)	4 (7.84)	0.009
Pakistan	32 (32)	31 (31)	16 (16)	16 (16)	5 (5)	
